# A Method for Checking Genomic Integrity in Cultured Cell Lines from SNP Genotyping Data

**DOI:** 10.1371/journal.pone.0155014

**Published:** 2016-05-13

**Authors:** Petr Danecek, Shane A. McCarthy, HipSci Consortium, Richard Durbin

**Affiliations:** Wellcome Trust Sanger Institute, Wellcome Trust Genome Campus, Cambridge, CB10 1SA, United Kingdom; Hospital Authority, CHINA

## Abstract

Genomic screening for chromosomal abnormalities is an important part of quality control when establishing and maintaining stem cell lines. We present a new method for sensitive detection of copy number alterations, aneuploidy, and contamination in cell lines using genome-wide SNP genotyping data. In contrast to other methods designed for identifying copy number variations in a single sample or in a sample composed of a mixture of normal and tumor cells, this new method is tailored for determining differences between cell lines and the starting material from which they were derived, which allows us to distinguish between normal and novel copy number variation. We implemented the method in the freely available BCFtools package and present results based on induced pluripotent stem cell lines obtained in the HipSci project.

## Introduction

Induced pluripotent stem (IPS) cells can be generated from adult tissues and differentiated into specific cell types. The reprogramming process involves clonal selection and cell line passaging, and is known to accumulate genomic aberrations, such as point mutations (estimated six protein-coding mutations per cell line on average), de novo copy number variations (CNVs) and aneupoloidy [1–4]. These abnormalities are often not detectable by classical karyotyping due to the limited sensitivity of the technique [5]. More sensitive detection methods based on array genotyping platforms have been developed in the past years. Although arrays designed for CNV detection are available, standard single nucleotide polymorphism (SNP) genotyping arrays designed for association studies can also successfully be used [6]. Despite limitations in sensitivity dictated by the spacing of markers, the genotyping chips are practical for routine detection of novel CNVs when compared to next-generation sequencing because of their low cost, high throughput and small amounts of DNA required.

**Fig 1:**
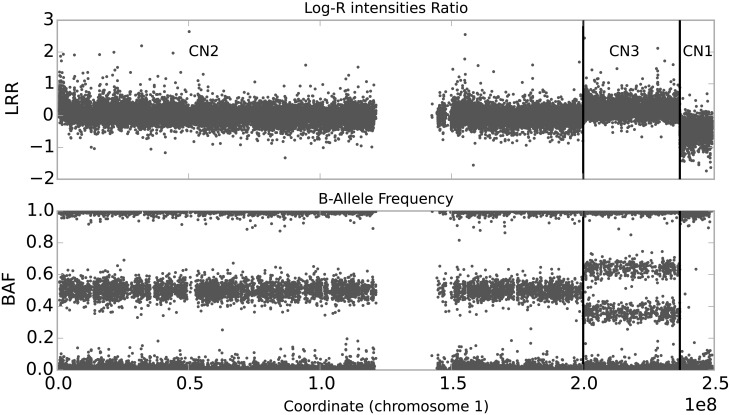
An example of an aberrant cell line with a duplication (CN3) and a deletion (CN1) on chromosome 1. Each dot in the graphs represents a single marker, the gap in the middle corresponds to a centromere, which is not targeted by the chip. The top graph shows the LRR values, which are centered around 0 in diploid regions (CN2), elevated in duplicated regions (CN3), and lowered in deletions (CN1). The bottom graph shows the corresponding BAF values which cluster into three bands in diploid regions, into four in CN3 regions, and into two in CN1 regions, as explained further in the text.

Genotype array data provides measurements at each of hundreds of thousands of genetically variable sites. The raw measurements consist of two intensity signals, one for each allele, which are subsequently transformed into the log-scaled ratio of the observed and the expected intensity (LRR), and the B Allele Frequency (BAF) which captures the relative contribution from one allele (B) to the fluorescent signal. An example is given in Fig 1.

A number of free and commercial programs have been developed for detecting CNVs which employ t-tests and standard deviations of the LRR values [7], segmentation methods [8] or hidden Markov models (HMM) [9, 10]. Several comparison studies attempted to evaluate the most reliable callers, concluding that there were large variations in both the number and the length of CNVs detected by different methods [11, 12]. This was true even for programs using the same methodology (HMM) and the same input data (BAF and LRR values). This uncertainty reflects not only the inherently noisy nature of the experimental data but also lack of robustness of the methods, where a small change in model parameters can have a significant impact on the results.

Although determining the absolute copy number state can be challenging, the problem of cell line screening is easier. Here, we are interested in detecting differences between a cell line and an independent control sample from the same individual from which the cell line was made, for example to screen out lines with major genomic aberrations. This problem is similar to that of cancer, where data from a tumour sample is compared to a control from non-cancerous tissue. A number of sophisticated algorithms have been developed for this type of problem [13–15] which are able to model the copy number state even for a sample composed of a mixture of normal and tumor cells. Some of these methods can take normal tissue as an optional input, however they do not take into account normal copy number variation in the control and assume diploidy [13].

In this paper, we present a method for screening cultured cell lines for genomic abnormalities. The method consists of two programs implemented in BCFtools [16]. The first, BCFtools/polysomy command, is intended for initial screening to identify contamination and cell lines with whole chromosome aberrations, namely aneuploidy. The second, BCFtools/cnv command, detects relative copy number variation local to a region of a chromosome. The program and source code are freely available at http://github.com/samtools/bcftools.

## Methods

### Aneuploidy and contamination

**Fig 2:**
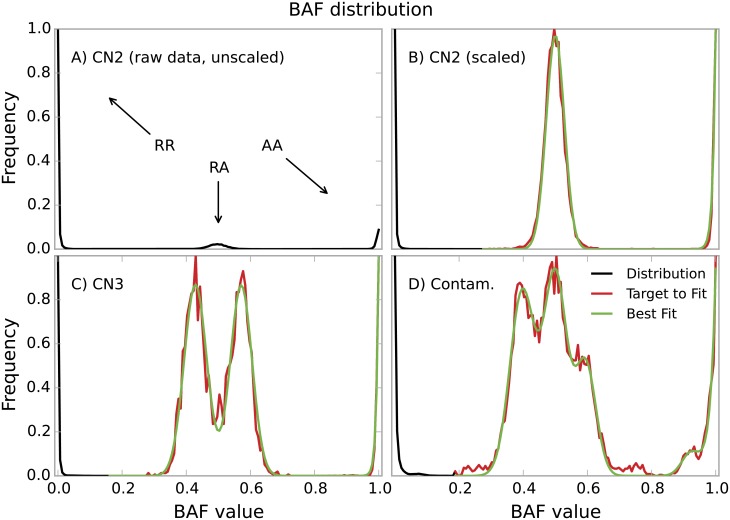
Input data for the polysomy method. Unscaled (A) and scaled (B-D) distributions of BAF values typical for the copy number states 2-4. In (C) we infer that 33% of the cells are aneuploid copy number 3, and in (D) we infer a sample with 20% contamination. The black line is the complete BAF distribution over 0 to 1 of which only part is modelled (shown in red); the green line is the best fit to the red part of the distribution. The model does not include the RR peak and including the AA peak is optional.

Large aberrations which affect whole chromosomes, such as aneuploidy or contamination, can be discerned directly from the overall distribution of BAF values (Fig 2). In the normal copy number state with two chromosomal copies (CN2), the distribution has three distinct peaks which result from the three possible genotypes: both copies can carry two reference alleles (RR), two alternate alleles (AA), or one reference and one alternate allele (RA). Because RR genotypes are more frequent than RA and AA genotypes, the RR peak dominates the distribution and it is practical to work with scaled RA and AA peaks (compare Fig 2A and 2B). This is done by smoothing the distribution using a moving average, finding local minima to identify peak boundaries, and normalizing the peak heights. Trisomies (CN3) can be identified by the presence of two central peaks which result from the RRA and RAA genotypes (Fig 2C). Their distance from the center gives an estimate of the proportion of aberrant cells, thus if *f* is the fraction of affected cells, the peaks are centered around 1/(2 + *f*) and (1 + *f*)/(2 + *f*). Finally, the BAF distribution of a contaminated sample has one central RRAA peak and two RRRA and RAAA side peaks symmetrical in position (but asymmetrical in intensity) when contamination is strong, or several peaks in the case of partial contamination (Fig 2D). The distance between the side peaks can again be used to estimate the extent of the contamination.

In order to determine the copy number state, the method employs the Levenberg-Marquardt algorithm [17] to perform a nonlinear least squares fit of the expected theoretical distribution to the observed distribution. We assume normally distributed BAF values and therefore attempt to fit one or more Gaussian peaks. Because heterozygous genotypes in the central part of the distribution are sufficient for determining the copy number state, the RR peak is not included in the fitting and including the AA peak is optional. The symmetry of the problem also enables the application of the following constraints: the RA peak is centered around 0.5; the RRA and RAA peaks are placed symmetrically around 0.5 and have the same magnitude; also the RRRA and RAAA peaks are placed symmetrically around 0.5 but their magnitudes can differ according to the allele frequency distribution of the genotyped markers.

The fitting is performed individually for each copy number state and then the most likely state is selected according to the goodness of fit. Because multiple peaks are always an equal or a better fit to the experimental data than a single peak, an aberrant copy number state is accepted only if the absolute deviation of the fit to the data is smaller than a given percentage of the single peak deviation (the default is 30%). When the data deviate strongly from the expected distributions or none of the alternatives is markedly better than the others, the program reports a failure to assign the copy number state.

Finally, an additional constraint is put on the minimum fraction of aberrant cells to assure clean separation of the side peaks, which helps to reduce the number of false calls with noisy data. As shown in Results, a practical threshold is 20% but this can be set higher when higher specificity is required.

### CNV detection

For smaller localised copy number variations we employ HMMs similarly to the work of others [10], with differences explained below. The basic HMM models four copy number states: normal diploid (CN2), loss (CN1 or CN0), and a single-copy gain (CN3). We explored the use of a separate copy number four (CN4) state but it proved hard to parametrize such model to generate stable separation from the CN3 state, for example in trisomies. For our primary aim of screening the integrity of cell lines it is not necessary to distinguish CN4 from CN3, since both are abnormal.

The Viterbi algorithm is used to find the most likely copy number state in a region and the average posterior probability calculated by the forward-backward algorithm is used to assign a quality score to each. We also build a two-sample HMM with state space the product of the basic HMM for both samples (16 states) and favoring joint transitions to keep the states the same except where there are mutations. This has the desired effect of ignoring population variation shared by both samples.

**Fig 3:**
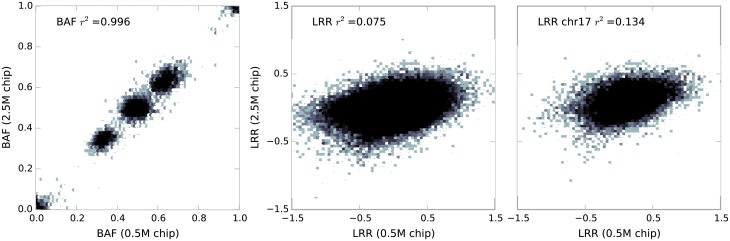
The correlation of BAF and LRR values obtained in the validation experiment using the default chip (0.5M sites) and the high density chip (2.5M sites) on sample HPSI0713i-virz_2_QC1Hip. The central plot shows LRR correlation across the whole genome. The right hand plot shows LRR correlation across chromosome 17 which contains a large 40Mb duplication. The plots are 2-D histograms with hexagonal bins, logarithmic greyscale was used to indicate the number of markers in a bin.

Following the assumption that the BAF values are normally distributed, we set the probability of observing the BAF value *β* (0 ≤ *β* ≤ 1) in the copy number state *s* as

$$\begin{array}{cc} P(\beta |s=0) & =0\\ P(\beta |s=1) & =G\left(\beta ,0\right)\;\left(f_{RR}+0.5f_{RA}\right)+G\left(\beta ,1\right)\;\left(f_{AA}+0.5f_{RA}\right)\\ P(\beta |s=2) & =G\left(\beta ,0\right)\;f_{RR}+G\left(\beta ,1\right)\;f_{AA}+G\left(\beta ,0.5\right)\;f_{RA}\\ P(\beta |s=3) & =G\left(\beta ,0\right)\;f_{RR}+G\left(\beta ,1\right)\;f_{AA}+\left[G(\beta ,1/3)+G(\beta ,2/3)\right]\;0.5f_{RA} \end{array}$$ (1)

where *f*_*RR*_ (resp. *f*_*RA*_, *f*_*AA*_) is the prior probability of the RR (resp. RA, AA) genotype calculated from site allele frequencies, and

$$\begin{array}{c} G\left(\beta ,\beta_{0}\right)=c_{\beta_{0}}\;e^{-{\left(\beta -\beta_{0}\right)}^{2}/d^{2}} \end{array}$$ (2)

is the probability of observing the value *β* instead of the expected value *β*_0_. The scaling factor *c*_*β*_0__ in this equation ensures that the area under each peak scales to one and *d* is the standard deviation which accounts for noise in the data and by default is set to 0.04 [18]. It is also assumed that LRR is normally distributed and the probability of observing the LRR value *λ* is

$$\begin{array}{c} P\left(\lambda |s\right)\propto e^{-{\left(\lambda -\mu_{s}\right)}^{2}/\Lambda^{2}}, \end{array}$$ (3)

where *μ*_*s*_ is -0.45, 0, 0.3 for the copy number states *s* = 1, 2, 3 as in [18] and the default standard deviation Λ is set to 0.2. The emission probability is then expressed as

$$\begin{array}{c} P\left(\beta ,\lambda |s\right)=P_{err}+\left[1-b\;(1-P(\beta |s))\right]\left[1-l\;(1-P(\lambda |s))\right], \end{array}$$ (4)

where *P*_*err*_ is a uniform probability of an erroneous reading, and *b* and *l* (0 ≤ *b*, *l* ≤ 1) moderate the contribution from BAF and LRR respectively. This feature is motivated by the observation that the LRR values are less reliable and offer a poor signal-to-noise ratio compared to the BAF values (see Results section and Fig 3).

Sites at which no call was made by the genotyping software may derive from the locus being missing entirely in the sample (*s* = 0), or from a technical failure. These are ignored in single sample calling, but are considered in two-sample calling. In that case we set arbitrarily *P*(*β* = N/A|*s* = 0) to 0.5 and *P*(*β* = N/A|*s* = 1, 2 or 3) to 0.5/3 each.

In the case of two-sample calling, the emission probability is

$$\begin{array}{c} P\left(\beta_{1},\lambda_{1},\beta_{2},\lambda_{2}|s_{1},s_{2}\right)=P\left(\beta_{1},\lambda_{1}|s_{1}\right)\;P\left(\beta_{2},\lambda_{2}|s_{2}\right). \end{array}$$ (5)

The HMM transition matrix *T* for single sample calling is symmetric, with all off-diagonal elements set equally to *p*_*ij*_ = *P*(*i*|*j*) = *p* where 0 < *p* < 1/3, and diagonal elements set to *p*_*ii*_ = *P*(*i*|*i*) = 1−3*p*. The probability of transition *p* is customizable and the default value 10^−9^ was used in this study. For pairwise calling, we introduce the parameter *σ* (0 ≤ *σ* ≤ 1) which controls the prior probability of the two samples being in the same copy number state. The probability of the first sample making the transition *i* → *j* and the second simultaneously making the transition *x* → *y* is set as

$$\begin{array}{c} P\left(jy|ix\right)=\left\{\begin{array}{cc} p_{jj}\;p_{ii}\left(1-\sigma \right)+\sigma \;\sqrt{p_{jj}\;p_{ii}} & \text{if}\;j=y\;\text{and}\;i=x,\\ p_{ji}\;p_{jx} & \text{if}\;j=y,\\ p_{ji}\;p_{yx}\left(1-\sigma \right) & \text{otherwise}. \end{array}\right. \end{array}$$ (6)

Then these values are normalized so that ∑_*ix*_ *P*(*jy*|*ix*) = 1.

In order to account for the fact that the markers are unevenly distributed on chromosomes, the transition matrix *T* is updated at position *j* according to the distance from the previously encountered marker *i* so that *T*(*i*, *j*) = *T*^*j*−*i*^. In practice we pre-calculate the matrix for 10,000 positions and any gaps in the data which are longer are calculated dynamically.

The HMM states are initially set to prefer the normal copy number state CN2: *P*(*s* = 2) = 0.5 and *P*(*s* ≠ 2) = 0.5/3 for the basic HMM and the product of these for the two-sample HMM.

#### Sensitivity of CN3 detection

The calculation of emission probabilities relies on several parameters. Some are general statistical descriptors that can be readily calculated from the data, such as the standard deviation *c*_2_ in Eq 2. However, other parameters must be estimated iteratively. Specifically, the expected BAF values *β*_0_ depend on the fraction of aberrant cells in the mixture, but estimating this depends on correctly assigning non-diploid copy regions. When only a fraction of cells is aberrant, the most likely BAF value *β*_0_ shifts towards 0.5 for heterozygous genotypes (S1 Fig), which can result in missed calls because the emission probability of the CN3 state can become as low as that of CN2, or lower. Therefore, in addition to the default *β*_0_ which assumes that all cells are aberrant, the program can also attempt to optimize the parameter from the data. Because of the symmetry of the problem, only the RRA genotype needs to be considered. A new estimate is calculated from the posterior probabilities *P*_*i*_(*s* = 3) at all heterozygous sites *i* as

$$\begin{array}{c} \beta_{0}^{\prime }=\frac{\sum_{i}(0.5-|0.5-\beta_{i}\left|\right)P_{i}\left(s=3\right)}{\sum_{i}P_{i}\left(s=3\right)}, \end{array}$$ (7)

where *β*_*i*_ is the BAF value observed at *i*-th site. This step is performed iteratively until $\beta_{0}^{\prime }$ converges, or until $0.5-\beta_{0}^{\prime }$ becomes too small to keep 95% of normally distributed BAF values smaller than 0.5. This condition ensures a sufficient separation for the two heterozygous genotypes and dynamically adjusts itself according to the level of noise encountered in the data.

## Results

The performance of the methods was evaluated on simulated data and compared to the popular PennCNV caller.

The programs were then applied to genotyping data from cell lines generated in the HipSci project [19] as part of the quality control pipeline. The data were obtained using the Illumina HumanCoreExome array which tests 270k coding variants and 250k genome-wide variants. In order to evaluate the performance and test the reliability of the experimental intensities, 48 cell lines were re-genotyped on a higher-density chip (Illumina Omni2.5-8exome, 2.5 million markers). The samples in this main validation experiment included all aberration types we typically observe in cultured cell lines: 32 were selected because of duplications, losses, and trisomies; 8 because of various levels of suspected contamination; and 8 to assess the reproducibility of an increased LRR and BAF variation occasionally observed in some samples. For cost efficiency, the samples were chosen so that multiple aberration types could be tested at once. For example, the selection included samples with a trisomy on one chromosome and smaller duplications on other chromosomes, or samples with suspected low levels of contamination and several deletions at the same time. Thus we explicitly note that the sample selection was non-random and strongly biased towards less obvious cases, with the primary aim to determine thresholds for confident acceptance or rejection of a cell line, rather than to quantitate the performance of the methods.

Unless explicitly stated otherwise, both programs were run with default parameters. PennCNV was tested with both its hh550 and hhall setups, hhall performed better and was used in the final analysis. The allele frequency estimates (-pfb) were based on the frequencies observed in the HipSci samples. When run on a single simulated chromosome, PennCNV was used with the -nomedianadjust option.

### Input Data Reliability

As shown in Fig 3, the BAF quantity was reproducible to a high degree (*r*^2^ = 0.997), however, LRR values appeared almost uncorrelated (*r*^2^ = 0.075). This is not completely unexpected, because most of the measurements come from markers in the normal copy number state where LRR values are distributed randomly around 0, whereas BAF values are scattered around 0, 0.5, and 1. After restricting the calculation to chromosomes with significant aberrations (e.g. half a chromosome duplicated), the LRR correlation improved, but nonetheless remained low (*r*^2^ = 0.134), irrespective of the sample and the reason for including the sample in the validation set.

In cases where the main source of the LRR variation is random statistical noise, the data can be cleaned by applying a moving average [6] or other low-pass filtering technique. The correlation can be improved up to *r*^2^ = 0.7 in such data (S2 Fig), but the smoothing cannot correct strong systematic biases such as shown in S3 Fig.

Based on these observations we can expect the CNV calling method to be less robust than the polysomy method, because it takes as input both BAF and LRR values. Also rather than working with whole distributions of values, it takes into consideration signals from each marker individually, making it even more sensitive to input data noise. Therefore, less significance was given to the relative contribution of LRR when calling from real data. The default values (*b* = 1, *l* = 0.2 in Eq 4) were chosen to capture all large aberrations observed in a training set of randomly selected samples.

### Simulated data

The simulations focused on the CNV calling method with the aim to determine the specificity and the sensitivity of the caller under different conditions.

The size limit for detecting CNVs is dictated by spacing of the markers on the genotyping array (the median of which is 2kb in the case of the 0.5M CoreExome array) and the percentage of segregating heterozygous genotypes (which was 15% in our data). The simulated genotypes were assigned to the markers randomly according to RR, RA and AA frequencies observed in real data. Signal intensities were then sampled from a normal distribution using variation observed in real data, which ranged from 0.03 to 0.08 for BAF and from 0.13 to 0.26 for LRR. Ten thousand simulated experiments were performed, producing 23,033 simulated aberrations which ranged from 18kb to 4Mb (1st and 99th percentile) with median 1.7Mb. The false positive and false negative counts were calculated based on overlaps of arbitrary length. In addition, the missed length and the length of falsely called regions was analysed.

**Fig 4:**
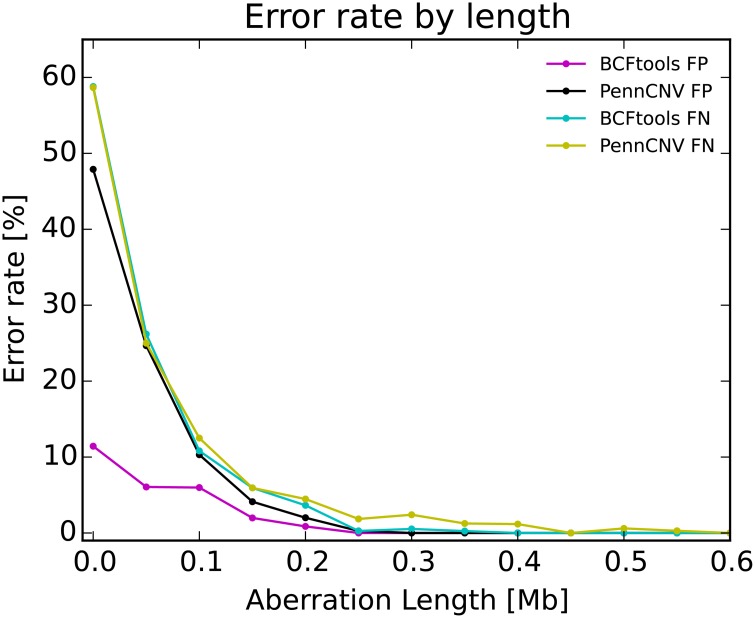
Error rate by length in simulated data.

Of the 23,033 simulated regions, BCFtools and PennCNV failed to recognise 1.7% and 1.8%, respectively. After the raw PennCNV calls were cleaned using its recommended post-processing script, the number of missed calls decreased to 1.6%. The false discovery rate was 0.3% for BCFtools, 1.2% for PennCNV and 1.1% for the cleaned PennCNV calls. In general, duplications were more difficult to call and formed a larger fraction of both false negatives (BCFtools 55%, PennCNV 73%) and false positives (BCFtools 100%, PennCNV 95%). BCFtools required at least four markers to call a deletion, capturing 99.9% deletions which spanned ten markers or more and 98.8% duplications which spanned four heterozygous markers or more (S4 Fig). Most miscalled regions were smaller than 500kb and the error rate significantly increased in regions smaller than 200kb although the false positive rate increased more slowly for BCFtools than for PennCNV (Fig 4).

BCFtools was more robust to input data noise and the error rate remained relatively constant throughout the whole range of LRR and BAF values (S5 Fig, top row). Also the region boundaries were detected with greater precision by BCFtools and the use of the post-processing script was required for PennCNV (S5 and S6 Figs, bottom row).

### High-density array data

#### The polysomy method

**Fig 5:**
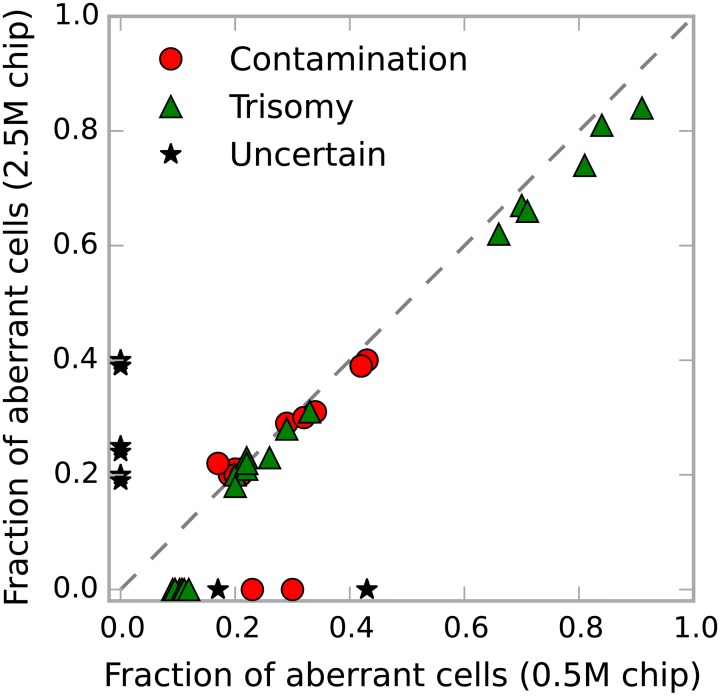
Reproducibility of polysomy results across different chips. Fraction of contaminating cells in the sample (red circles) and the fraction of cells with trisomies (green triangles) as estimated from the default (0.5M sites) and higher density chip (2.5M sites). All outliers in this figure are unconfirmed contaminations and chromosomes failing goodness of fit criteria as explained in the text.

The set of 48 samples included 12 trisomies which were all called also from the 2.5M Omni array data. The estimates of trisomy frequencies ranged from 18% to 91% of affected cells and were highly consistent (Fig 5).

Of the 8 samples which were tested for signs of contamination, 4 were confirmed and 4 were not, possibly because the contamination occurred at a different stage of sample processing (see S7 Fig for examples). Note that several chromosomes in the contaminated samples were called incorrectly as trisomies or were marked as uncertain by the program (outliers in Fig 5). This happens when the three central peaks of the CN4 state are centered so close to each other that they can be modelled sufficiently well by two peaks or even a single broader peak (S7C Fig). In practice this does not constitute a problem because contaminations can be easily distinguished from trisomies by observing the same pattern on all chromosomes.

The estimates of contamination frequencies in the confirmed samples ranged from 10% to 43% of foreign cells, and were also highly consistent (Fig 5). The estimated levels for the unconfirmed samples ranged from 10% to 23%. We conclude that the method can be used to detect contamination and trisomies down to approximately 20-25% frequency.

#### The CNV calling method

Although we cannot estimate true false positive and false negative rates from our data, we can report the fraction of calls confirmed by the higher density chip. The median distance of the markers on the 2.5M array is 600bp which allows us to identify false calls caused by short runs of homozygous markers and check the consistency of the calls across two platforms. In the comparisons that follow, we exclude calls which overlap centromeres as they are not targeted by the arrays.

As expected, the CNV calling method was much less robust than the polysomy method when applied to real data. BCFtools made 315 calls of which 78.4% intersected with a call made from the 2.5M Omni array data. From the same data PennCNV made 7041 calls of which 61.9% were reproduced. We then performed the same test with QuantiSNP [9]: it made 3570 calls of which only 33.9% were reproduced. The big difference between the callsets and the lower reproducibility of PennCNV and QuantiSNP calls is caused by a greater willingness of PennCNV and QuantiSNP with its default HMM parameters to make multiple fine scale transitions between copy number states: 87% of the calls made by PennCNV and 76% of the calls made by QuantiSNP were smaller than 200kb, whereas only 37% of the calls made by BCFtools were (S8 Fig). It should be emphasized that these results should be interpreted carefully as they are highly dependent on program settings.

Many of the false calls can be effectively prevented by running BCFtools in pairwise mode, where a prior is set on the control and the query samples sharing the same copy number state. The effect of the prior is shown in S1 Table. For samples that are assumed independent (*σ* = 0, Eq 6), the number of called differences is largest and gradually decreases with the increasing value of *σ*, removing approximately one third of the calls at *σ* ≈ 0.5 where the calls are likely to reflect real differences between the two samples. When applied on 18 pairs which were present among the 40 non-contaminated samples, the confirmation rate of novel aberrations increased from 80.1% in the single sample mode to 90.0% in the pairwise mode with *σ* = 0.5.

BCFtools/polysomy and CNV have been used as part of the quality control pipeline for the HipSci project, which is making hundreds of human induced pluripotent cell lines (http://www.hipsci.org). A comprehensive analysis of all the aberrations detected in the HipSci cell lines will be presented elsewhere.

## Discussion

We have presented a method for checking genomic integrity in cultured cell lines using whole-genome SNP genotyping data. The method consists of two algorithms implemented in BCFtools/polysomy and BCFtools/cnv commands, the first for detection of whole chromosome aberrations and contamination, and the second for detecting sub-chromosomal aberrations.

The program was evaluated extensively and compared to the popular PennCNV caller. Both programs performed similarly well when run in single-sample mode on simulated data, with BCFtools having slightly lower error rates and being more robust against noise. However, while the performance was excellent on simulated data with correctly recognising 98.5% of CNVs larger than 10kb and 99.9% of CNVs larger than 200kb, both programs performed less consistently when applied to real data. The reproducibility of calls made from the two microarray platforms was 78.4% for BCFtools and 61.9% for PennCNV. Moreover, running BCFtools in pairwise mode increased reproducibility from 80.1% to 90.0%.

We found that the input data to these methods, BAF and LRR quantities, are affected by systematic and random noise to a different extent, LRR being much less reliable across different runs. Consequently, results from LRR-based methods tend to be less reproducible, unless precautions are taken. Our approach to this problem is to make the LRR contribution to the HMM emission probabilities less significant than the contribution from BAF. Also, in contrast to most other methods, the BCFtools/cnv command is tailored for determining differences between two cell lines, which increases robustness and, in addition, allows one to distinguish between normal and novel copy number variation.

We acknowledge that, as stated in the introduction, SNP genotyping arrays cannot detect all copy number variants, but they are cost effective and convenient for detection of large aberrations compared to custom CNV arrays or sequencing. We also note that, because of our focus on screening for genomic integrity, we did not attempt to resolve higher order copy number states. To our knowledge BCFtools/cnv is the first SNP-array based CNV caller for paired samples that explicitly models background copy number variation in the control sample. The resulting program has proved valuable for screening hundreds of cell lines in the HipSci project.

## Supporting Information

S1 File S1 File contains S1–S8 Figs and S1 Table. (PDF)

S2 File List of cell lines. (ODS)

S1 Text The full list of HipSci participants and their affiliations. (PDF)

S2 Text Laboratory methods. (PDF)

S1 Fig Example of CN3. A duplication (CN3) present in the majority of cells manifests in a wider split of heterozygous genotypes (**A**). The separation is less pronounced when a duplication is present in a smaller fraction of cells (**B**).
(TIFF)

S2 Fig LRR smoothing. The correlation between LRR values obtained from the default chip (0.5M sites) and the high density chip (2.5M sites) plotted as a function of the moving average window.
(TIFF)

S3 Fig Example of noise in LRR data. (TIFF)

S4 Fig Number of markers (for CN1) and heterozygous markers (for CN3) in regions missed by BCFtools and PennCNV. (EPS)

S5 Fig Robustness of the callers against input data noise. The top row shows the number of false positives and negatives as a function of increasing LRR and BAF noise. The bottom row shows the error in prediction of region boundaries: the FP curves show incorrectly added length to correctly detected regions and the FN curves show missed length from correctly detected regions.
(EPS)

S6 Fig Robustness of the callers against input data noise after PennCNV postprocessing. (EPS)

S7 Fig Example of three HipSci samples with unusual BAF distributions. The plots in the left column show BAF values from each marker individually and the plots on the right show the overall distribution of BAF values across whole chromosome. The top row (in red) of each panel shows the 0.5M array data, the bottom row shows the 2.5M Omni array data (black). The top sample (**A**) with estimated 20% contamination was confirmed by the higher density chip while the other two samples with estimated 21% and 15% contamination were not (**B** and **C**).
(TIFF)

S8 Fig Distribution of region lengths called by BCFtools, PennCNV and QuantiSNP. (EPS)

S1 Table The effect of *σ* in pairwise calling. The number and total length of novel CNVs observed across 905 cell lines from the HipSci project. No differences are allowed when *σ* = 1.0.
(PDF)
