# Supplementary material for: A Method for Checking Genomic Integrity in Cultured Cell Lines from SNP Genotyping Data
Source: PLoS One. 2016 May 13;11(5):e0155014. doi: 10.1371/journal.pone.0155014 (PMC4866717; doi:10.1371/journal.pone.0155014)
Supplement: S1 File — (PDF) [file pone.0155014.s001.pdf]

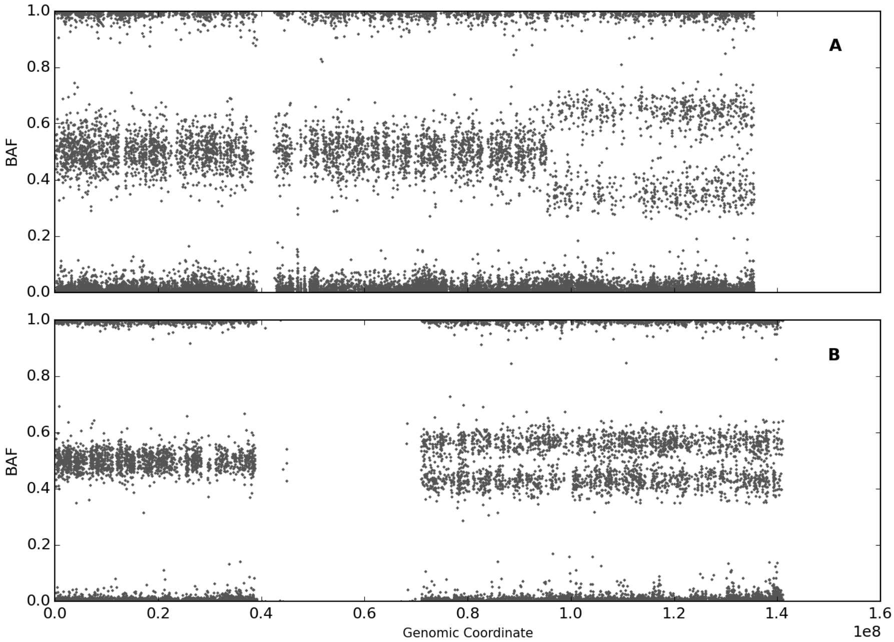

S1 Fig. Example of CN3. A duplication (CN3) present in the majority of cells manifests in a wider split of heterozygous genotypes (**A**). The separation is less pronounced when a duplication is present in a smaller fraction of cells (**B**).

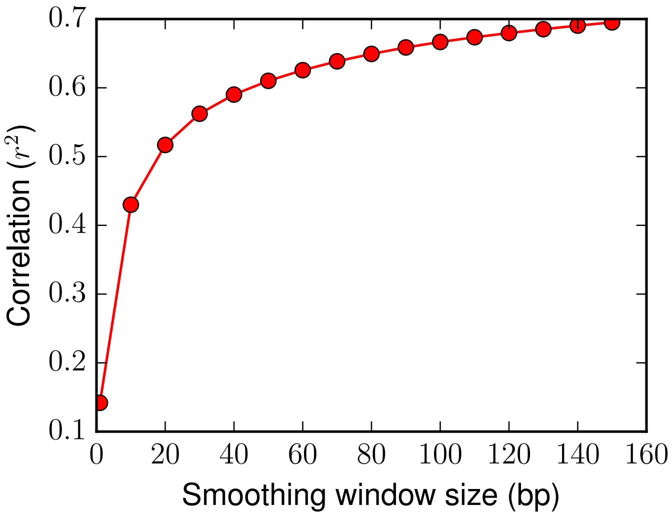

S2 Fig. LRR smoothing. The correlation between LRR values obtained from the default chip (0.5M sites) and the high density chip (2.5M sites) plotted as a function of the moving average window.

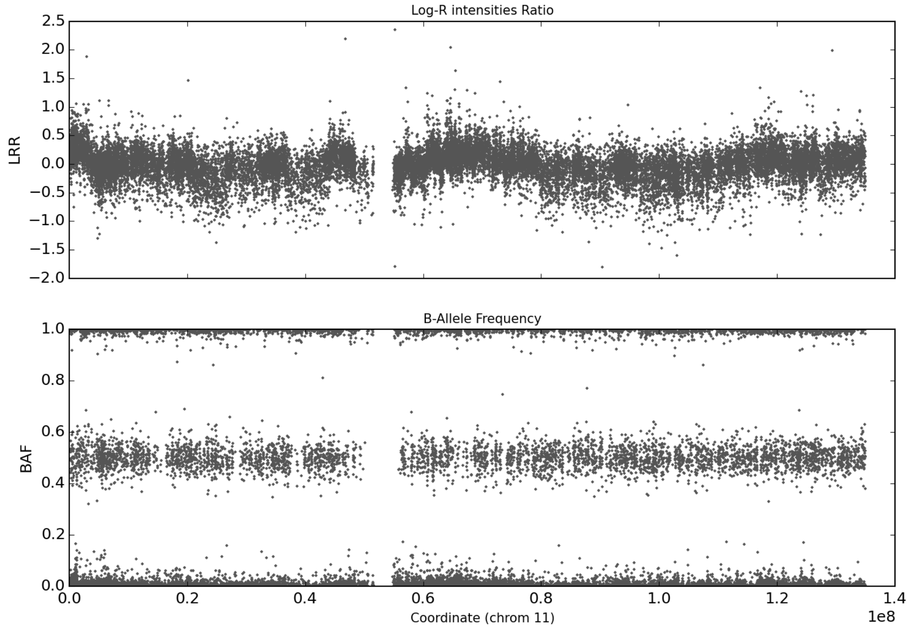

S3 Fig. Example of noise in LRR data.

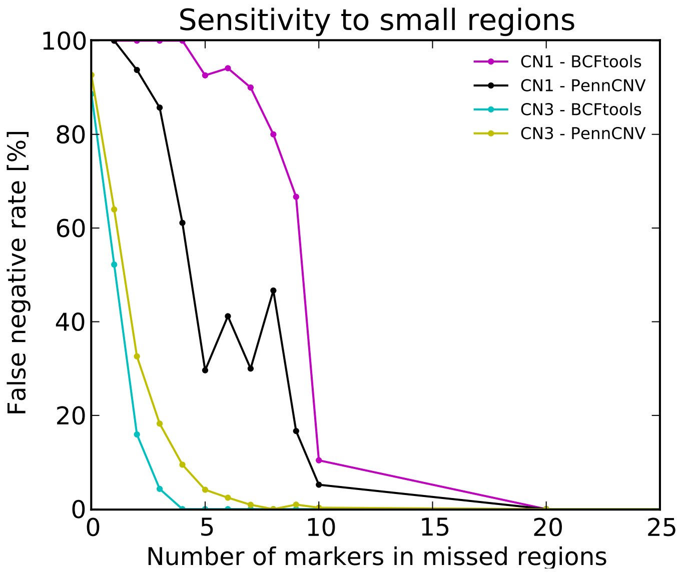

S4 Fig. Number of markers (for CN1) and heterozygous markers (for CN3) in regions missed by BCFtools and PennCNV.

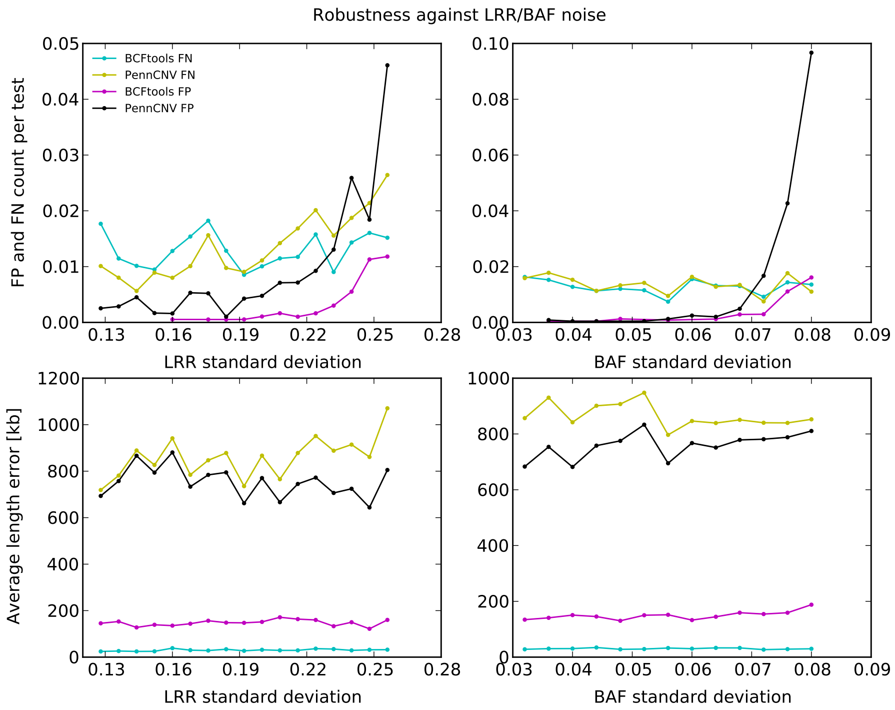

S5 Fig. Robustness of the callers against input data noise. The top row shows the number of false positives and negatives as a function of increasing LRR and BAF noise. The bottom row shows the error in prediction of region boundaries: the FP curves show incorrectly added length to correctly detected regions and the FN curves show missed length from correctly detected regions.

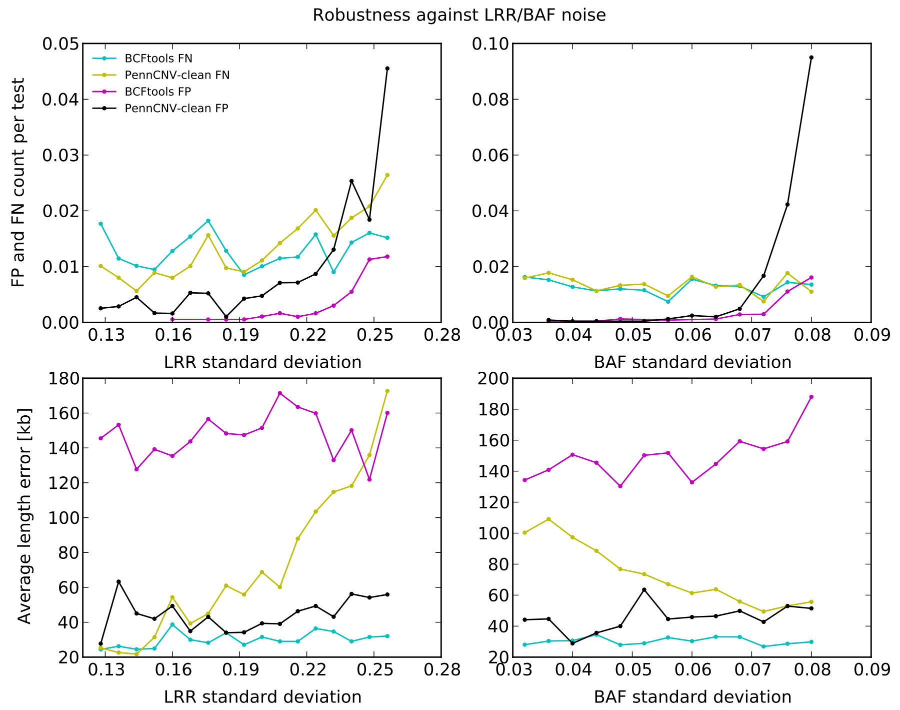

S6 Fig. Robustness of the callers against input data noise after PennCNV postprocessing.

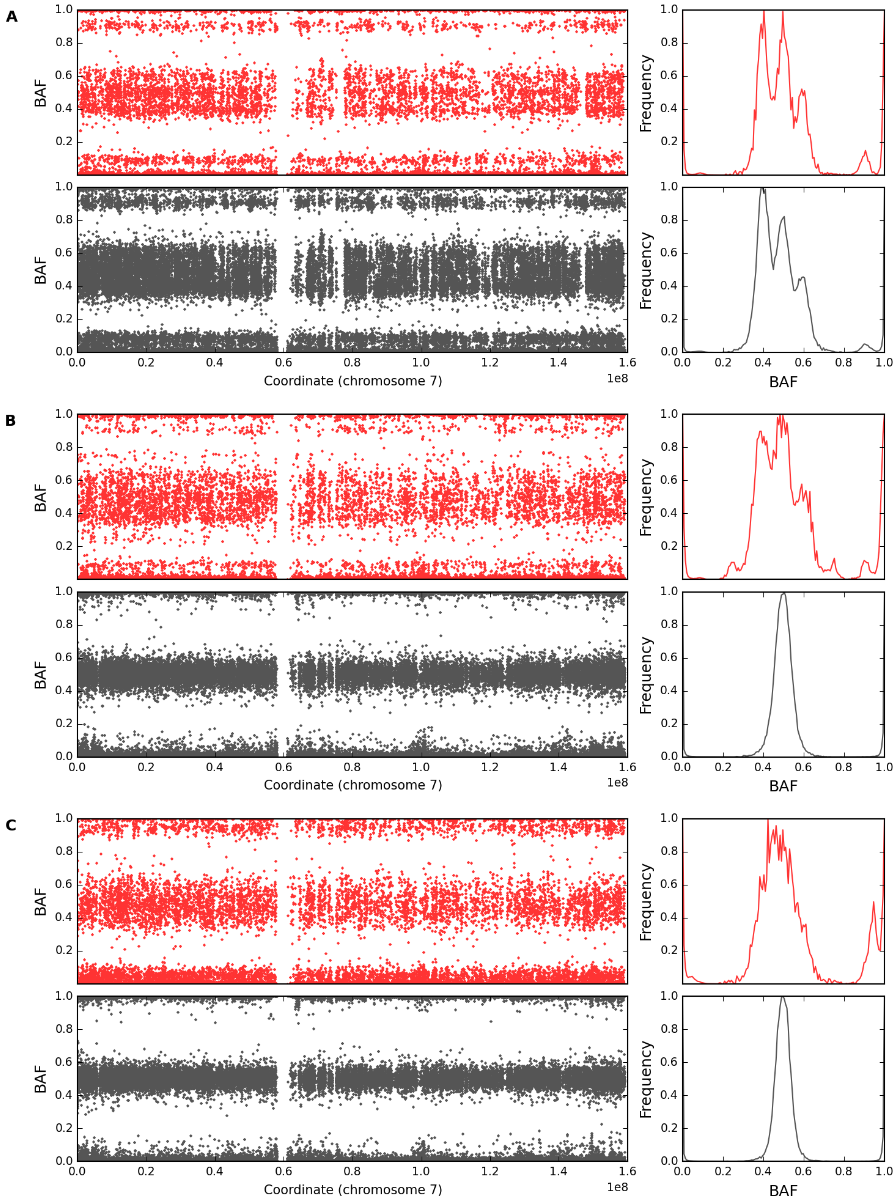

S7 Fig. Example of three HipSci samples with unusual BAF distributions. The plots in the left column show BAF values from each marker individually and the plots on the right show the overall distribution of BAF values across whole chromosome. The top row (in red) of each panel shows the 0.5M array data, the bottom row shows the 2.5M Omni array data (black). The top sample (**A**) with estimated 20% contamination was confirmed by the higher density chip while the other two samples with estimated 21% and 15% contamination were not (**B** and **C**).

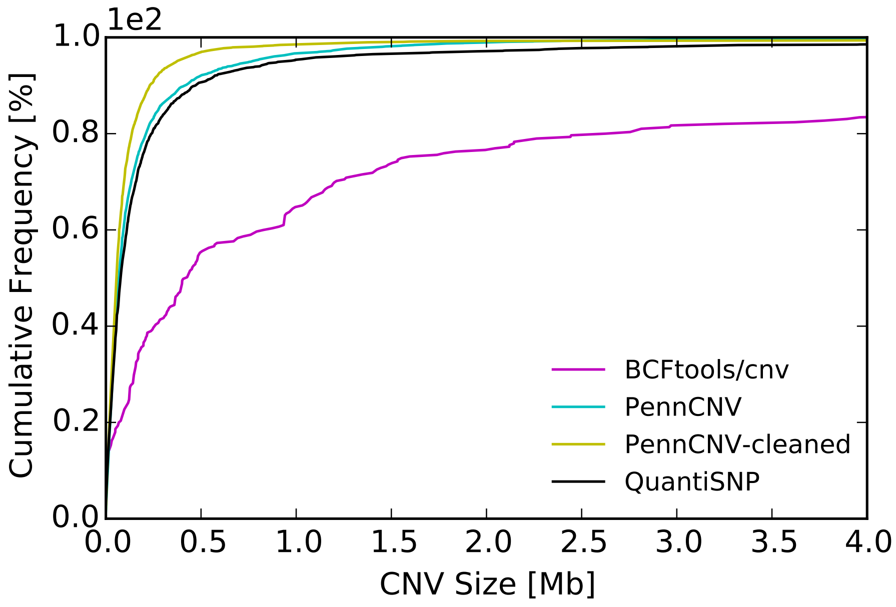

S8 Fig. Distribution of region lengths called by BCFtools, PennCNV and QuantiSNP.

S1 Table. The effect of σ in pairwise calling. The number and total length of novel CNVs observed across 905 cell lines from the HipSci project. No differences are allowed when $\sigma = 1.0$.

sameness prior σ	number of differences	total length of differences
0	1352	4900
0.0001	1006	4694
0.001	923	4603
0.01	882	4541
0.1	851	4504
0.5	831	4488
0.7	827	4487
0.8	824	4485
0.9	821	4485
0.99	819	4482
0.999	807	4464
0.9999	769	4346
