# Supplementary material for: A Method for Checking Genomic Integrity in Cultured Cell Lines from SNP Genotyping Data
Source: PLoS One. 2016 May 13;11(5):e0155014. doi: 10.1371/journal.pone.0155014 (PMC4866717; doi:10.1371/journal.pone.0155014)
Supplement: S1 Text — (PDF) [file pone.0155014.s003.pdf]

HipSci Consortium Author Information

Wellcome Trust Sanger Institute
Richard Durbin
Daniel Gaffney
Chukwuma Agu
Alex Alderton
Shrada Amatya
Petr Danecek
Rachel Denton
Angela Goncalves
Reena Halai
Sarah Harper
Chris Kirton
Andrew Knights
Anja Kolb-Kokocinski
Andreas Leha
Shane McCarthy
Yasin Memari
Minal Patel

European Molecular Biology Laboratory
Ewan Birney
Oliver Stegle
Francesco Paolo Casale
Laura Clarke
Peter Harrison
Helena Kilpinen
Davis McCarthy
Ian Streeter

King's College London
Fiona Watt
Davide Denovi
Ruta Meleckyte
Natalie Moens

University of Cambridge
Willem Ouwehand
Ludovic Vallier

University of Dundee
Angus Lamond
Dalila Bensaddek

University College London
Philip Beales
