# Supplementary material for: A Method for Checking Genomic Integrity in Cultured Cell Lines from SNP Genotyping Data
Source: PLoS One. 2016 May 13;11(5):e0155014. doi: 10.1371/journal.pone.0155014 (PMC4866717; doi:10.1371/journal.pone.0155014)
Supplement: S2 Text — (PDF) [file pone.0155014.s004.pdf]

Laboratory Methods

Author: Minal Patel

Cell cultivation Primary human fibroblasts were derived from 2mm skin punch biopsies. Fibroblasts derived from skin punch biopsies were cultured in fibroblast media consisting of Advanced DMEM, 10% FBS, 1% L-Glutamine, 0.007% 2-mercaptoethanol and 1% Pen/Strep, prior to reprogramming. IPSC derivation from human fibroblasts was performed using Sendai vectors expressing hOCT3/4, hSOX2, hKLF4, and hc-MYC (Takahasi and Yamanaka 2006). The transduced cells were maintained on and irradiated MEF-CF1 feeder layer in IPSC medium (Advanced DMEM (Life technologies, UK) supplemented with 10% Knockout Serum Replacement (KOSR, Life technologies, UK), 2 mM L-glutamine (Life technologies, UK) 0.007% 2-mercaptoethanol (Sigma-Aldrich, UK), 4 ng/mL of recombinant zebrafish basic Fibroblast Growth Factor-2 (CSCR, University of Cambridge), and 1% Pen/Strep (Life technologies, UK)). Undifferentiated colonies were picked and cultured on irradiated MEF feeder layers in iPSC medium using enzymatic (collagenase and dispase combined - Collagenase IV 1 mg/ml, Invitrogen 17104-019; Dispase 1 mg/ml, Invitrogen 17105-041) passaging. Cells were passaged every 5 to 7 days (depending on the confluence and morphology of the cells) at a maximum1:3 split ratio. IPSC lines were cultured to passage 3-5 before transferring to feeder free conditions.

Feeder free conditions consisted of Vitronectin-XF and E8 medium with EDTA passaging. IPSCs were cultured in Essential 8 (E8) medium on tissue culture dishes coated with 10ug/ml Vitronectin XF (StemCell Technologies, UK, 07180). E8 complete medium consists of basal medium DMEM/F-12(HAM) 1:1(Life technologies, UK, A1517001) supplemented with E8 supplement (50X) (Life technologies, UK, A1517001) and 1% Pen/Strep (Life technologies, UK, 15140122). Cells were passaged every 4-7 days (depending on the confluence and morphology of the cells) using split ratios ranging from 1:3 to 1:6.

Illumina Core Exome Beadchip Genotyping

Author: Michelle Dignam

Sample submission Samples are tested for quality, quantified and then normalised to 50ng/ul by the onsite sample management team prior to submission to the Illumina Genotyping laboratory. Where possible, samples are submitted in plates of 96 or multiples of 12 to reduce array loss/costs. Before processing begins, manifests for submitted samples are uploaded to Illumina LIMS where each sample plate is assigned an identification batch so that it can be tracked throughout the whole process.

Pre-Amplification On day one, pre-Amplification is performed using a Tecan Evo liquid handling robot to dispense four micro-litres (200ng) into a plate for whole genome amplification. This step happens overnight in an oven.

Post-Amplification Over three days, Post-Amplification (Fragmentation, Precipitation, Resuspension, Hybridisation to Beadchip and x-Staining) processes are completed as per Illumina protocol using the Tecan Freedom Evo Liquid

handling robot. Following the staining process, BeadChips are coated for protection and dried completely under vacuum before scanning commences on the Illumina iScans. Image Beadchip: The iScan Control software determines intensity values for each bead type on the BeadChip and creates data files for each channel (.idat). Genomestudio uses this data file in conjunction with the beadpool manifest (.bpm) to analyse the data from the assay.

Quality Control Prior to data release, all samples undergo an initial QC to establish how successful the assay has performed. Intensity graphs in Genomestudio's Control Dashboard identify sample performance by measuring dependant and non-dependant controls that are manufactured onto each beadchip during production.

Data release Raw intensities are provided in the form of .idat files and converted to .fcr files by the Genomestudio software.
