# Supplementary figures and images for: A Method for Checking Genomic Integrity in Cultured Cell Lines from SNP Genotyping Data
Source: PLoS One. 2016 May 13;11(5):e0155014. doi: 10.1371/journal.pone.0155014 (PMC4866717; doi:10.1371/journal.pone.0155014)

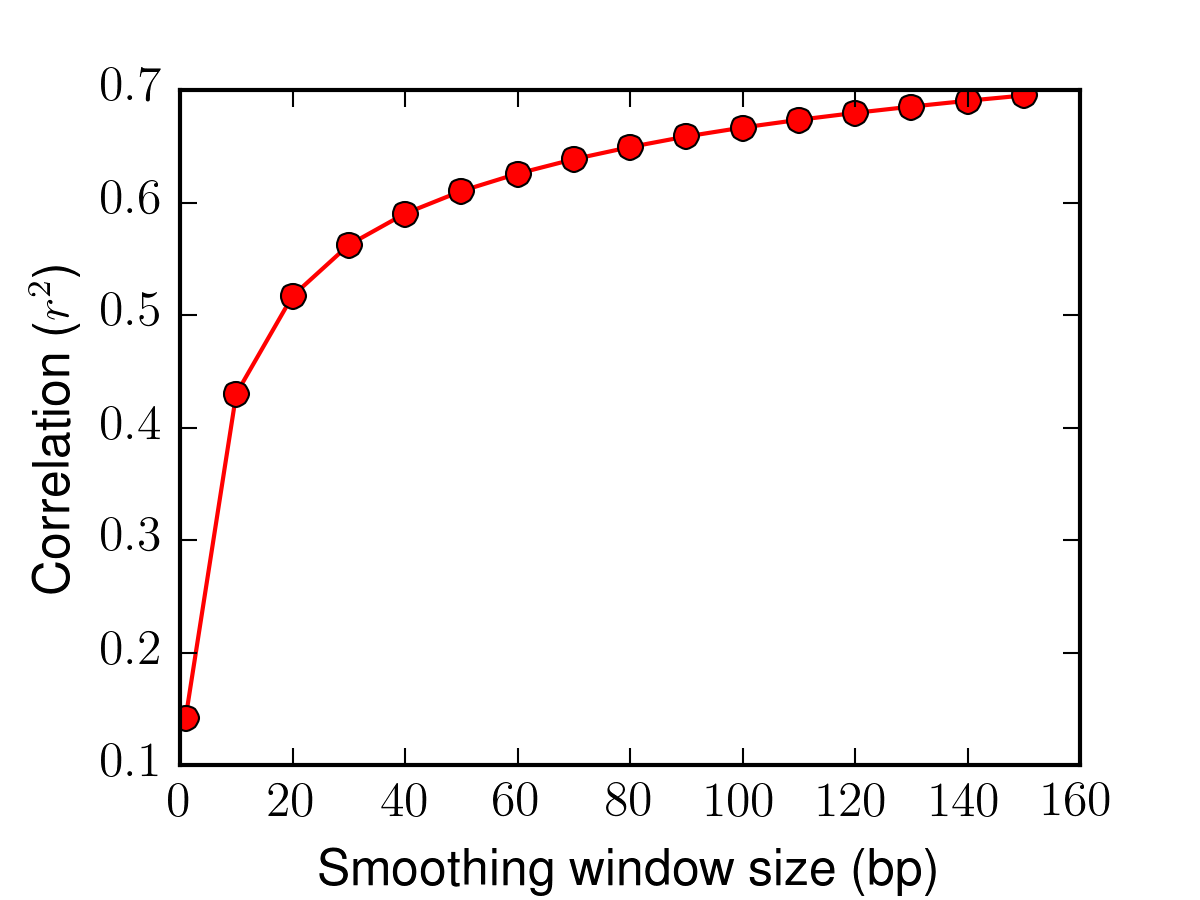

Supplement: S2 Fig — The correlation between LRR values obtained from the default chip (0.5M sites) and the high density chip (2.5M sites) plotted as a function of the moving average window. (TIFF) [file pone.0155014.s006.tiff]

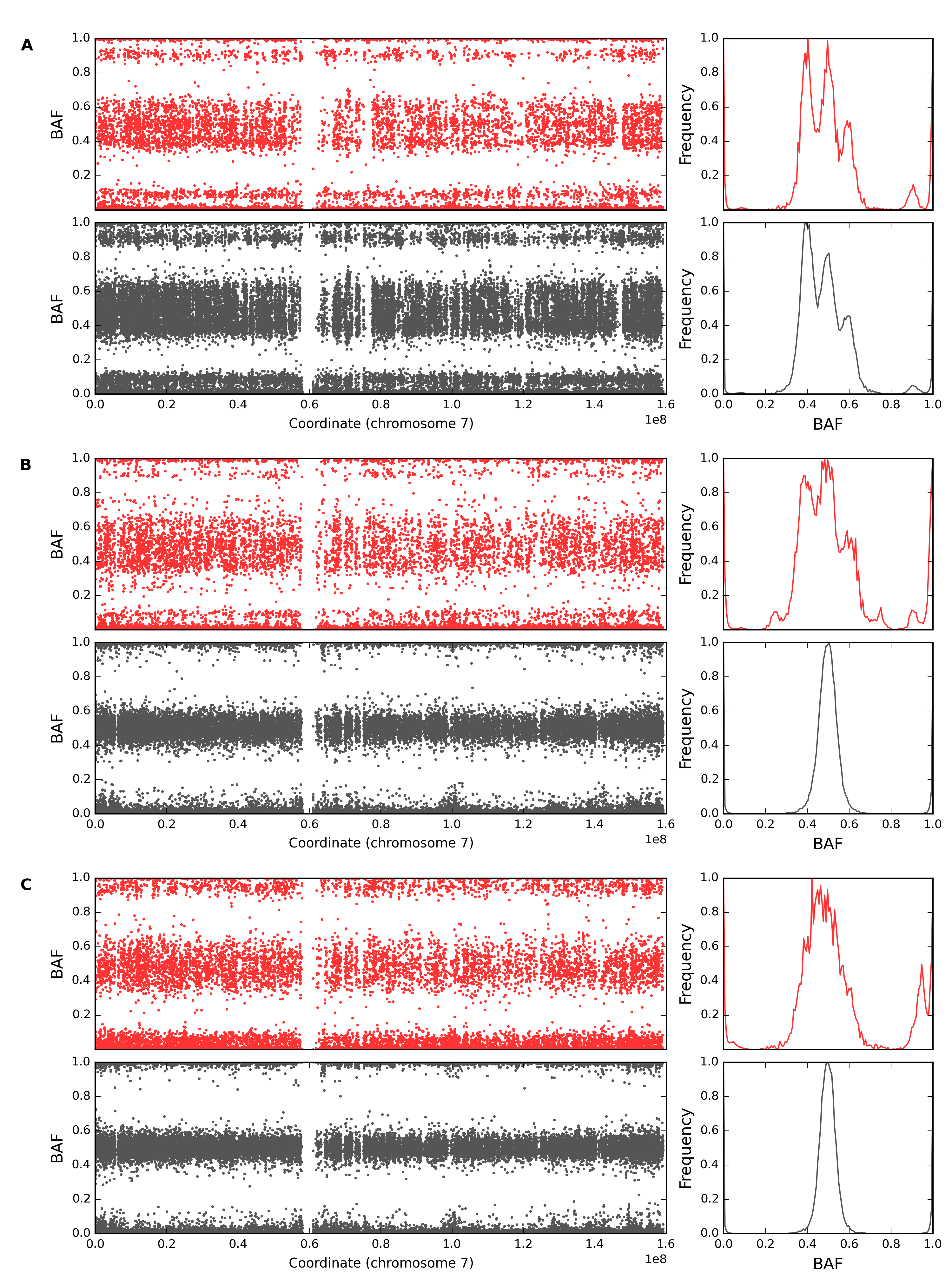

Supplement: S7 Fig — The plots in the left column show BAF values from each marker individually and the plots on the right show the overall distribution of BAF values across whole chromosome. The top row (in red) of each panel shows the 0.5M array data, the bottom row shows the 2.5M Omni array data (black). The top sample (A) with estimated 20% contamination was confirmed by the higher density chip while the other two samples with estimated 21% and 15% contamination were not (B and C). (TIFF) [file pone.0155014.s011.tiff]
