# Supplementary material for: A Method for Checking Genomic Integrity in Cultured Cell Lines from SNP Genotyping Data
Source: PLoS One. 2016 May 13;11(5):e0155014. doi: 10.1371/journal.pone.0155014 (PMC4866717; doi:10.1371/journal.pone.0155014)
Supplement: S1 Table — The number and total length of novel CNVs observed across 905 cell lines from the HipSci project. No differences are allowed when σ = 1.0. (PDF) [file pone.0155014.s013.pdf]

S1 Table. The effect of σ in pairwise calling. The number and total length of novel CNVs observed across 905 cell lines from the HipSci project. No differences are allowed when $\sigma = 1.0$.

sameness prior σ	number of differences	total length of differences
0	1352	4900
0.0001	1006	4694
0.001	923	4603
0.01	882	4541
0.1	851	4504
0.5	831	4488
0.7	827	4487
0.8	824	4485
0.9	821	4485
0.99	819	4482
0.999	807	4464
0.9999	769	4346
